# White Matter Hyperintensities and Cerebral Microbleeds in Ataxia-Telangiectasia

**DOI:** 10.1212/NXG.0000000000000640

**Published:** 2021-11-30

**Authors:** May Yung Tiet, Stefania Nannoni, Daniel Scoffings, Katherine Schon, Rita Horvath, Hugh Stephen Markus, Anke Erma Hensiek

**Affiliations:** From the Department of Clinical Neurosciences (M.Y.T., S.N., K.S., R.H., H.S.M.), University of Cambridge; Department of Radiology (D.S.), Addenbrooke's Hospital; and National Clinic for Ataxia Telangiectasia, Papworth Hospital NHS Foundation Trust (A.E.H.), Cambridge, United Kingdom.

## Abstract

**Background and Objectives:**

To systematically assess the occurrence of cerebral microbleeds (CMBs) and white matter hyperintensities (WMHs) in the largest published cohort of adults with ataxia-telangiectasia (AT).

**Methods:**

We assessed 38 adults with AT (age range 18–55 years) including 15 classic and 23 variant AT, evaluated by two independent assessors. WMHs were quantified on T2-fluid attenuated inversion recovery images using the semiquantitative modified Scheltens and Fazekas scales and CMB on susceptibility-weighted imaging and T2*-weighted gradient echo sequences using the Brain Observer MicroBleed Scale.

**Results:**

CMBs were more frequently found in classic AT compared with variant AT (66.7% vs 5.9%) predominantly in cortical and subcortical regions. WMHs were seen in 25 (73.5%) probands and CMBs in 9 (31.0%). The burden of WMHs increased with age, and WMHs were focused in periventricular and deep white matter regions. WMHs were more frequently seen in variant than classic AT.

**Discussion:**

This cohort study confirms that WMHs and CMBs are a frequent finding in AT. Further longitudinal studies are required to understand how WMHs and CMBs relate to the neurodegeneration that occurs in AT and the predisposition to cerebral hemorrhage.

Ataxia-telangiectasia (AT) is a rare autosomal recessive neurodegenerative DNA repair disorder that results in cerebellar ataxia and extrapyramidal symptoms.^1^ Although visceral telangiectasia-associated hemorrhages have been reported in AT, vascular abnormalities in the brain have not been systematically assessed.

Isolated small-scale studies suggest the presence of white matter hyperintensities (WMHs) and cerebral microbleeds (CMBs) in AT; both are associated with worse clinical outcomes in other neurodegenerative disorders, such as Alzheimer disease.^2^ It is important to identify potential cerebrovascular abnormalities in AT and how they relate to neurodegeneration. Here, we systematically analyzed our AT cohort for WMHs and CMBs.

## Methods

**Figure 1:**
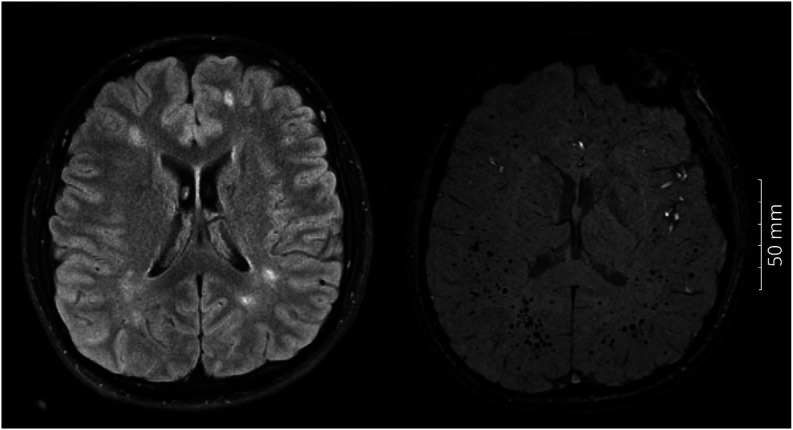
White Matter Hyperintensities (Left) in a Patient With Variant AT at Age 22 Years; Cerebral Microbleeds (Right) in a Patient With Classic AT at Age 23 Years.

From the Cambridge adult cohort, 38 subjects with confirmed AT (15 classic and 23 variant, age 18–55 years, 16 male and 22 female) underwent brain MRI scanning and were assessed for vascular risk factors and immunodeficiency (Figure 1). Two investigators independently analyzed T2-fluid attenuated inversion recovery for WMHs using the Fazekas and modified Scheltens score to quantify burden overall and individual brain regions.^3^ Susceptibility weighted imaging or T2*-weighted gradient echo sequences were reviewed for probable CMBs using the Brain Observer MicroBleed Scale.^4^ Images were excluded if movement artifact prevented systematic analysis.

Informed consent was obtained (13/YH/0310). Statistical analysis (Fisher exact and Mann-Whitney) was performed using GraphPad Prism 9.1.

### Data Availability

Anonymized data and documentation of this study will be shared on reasonable request from any qualified researcher. Standard data sharing agreements apply.

## Results

### Microhemorrhages Are More Frequent in Classic AT

**Figure 2:**
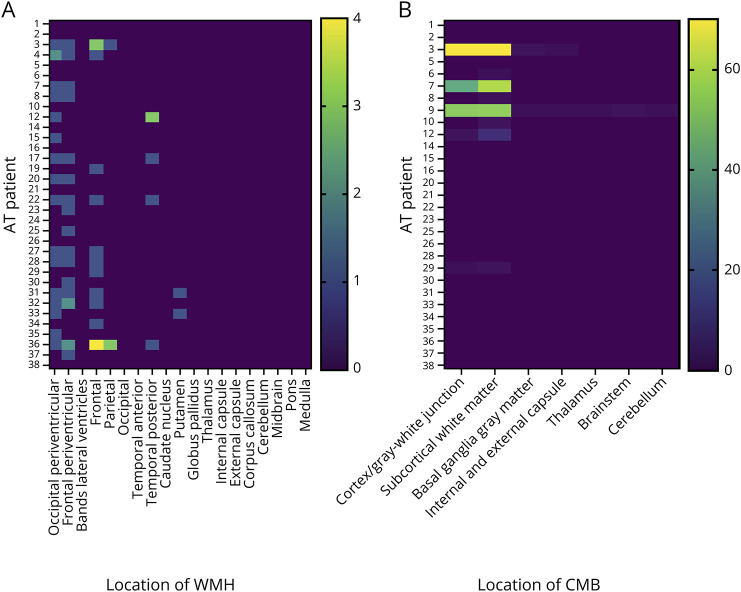
Heatmap Showing the Distribution of (A) White Matter Hyperintensities in Subjects With AT (Classic n = 12 and Variant n = 22). (B) Cerebral Microbleeds in Subjects With AT (Classic n = 12 and variant n = 17) AT = ataxia-telangiectasia.

Twenty-nine subjects (12 classic and 17 variant) were included in the CMB analysis. Nine subjects were excluded because of motion artifact or no susceptibility weighted imaging. CMBs were more commonly seen in classic AT (8 [66.7%] vs 1 [5.9%] [*p* < 0.01, OR 32.0, CI 3.17–369.8]). CMBs were commonly seen in cortical and subcortical regions rather than cerebellar and basal ganglia (Figure 2B).

Three subjects with classic AT had extensive CMBs (>100) (Figure 1). One patient who had extensive CMBs died of an intracerebral hemorrhage (ICH).

### White Matter Hyperintensities in AT

Thirty-four subjects (12 classic and 22 variant AT) were assessed for WMHs. Three probands were excluded because of motion artifact or lack of fluid attenuated inversion recovery sequencing and 1 because of extensive cerebral edema. Our subjects had only mild WMHs (Fazekas: mean 0.32, range 0–1, SD 0.47; modified Scheltens criteria: mean 2.29, range 0–11, SD 2.46).

WMH (Figure 1) was more frequent in variant AT (81.8% vs 58.3% classic, n = 18 and 7, respectively) but was not statistically significant (*p* = 0.224, OR 0.311, 95% CI 0.079–1.634). WMH burden increased with age (not shown) and was predominantly extracerebellar (Figure 2A).

### Genotype-Phenotype Correlations

A common leaking splice site variant was present in 26.1% (n = 6) subjects with variant AT, c.5763-1050A>G; p.Pro1922fs. No distinguishing phenotype was noted, but no patients with this variant had a CMB. A total of 8 subjects had cancer, and the presence of WMH was not associated with chemotherapy use (eTable 1, links.lww.com/NXG/A491).

The average age was 21.4 years for classic (n = 15) and 35.8 years for variant AT (n = 23) (Table). The body mass index, diabetes, and hypercholesterolemia were not significantly associated with WMHs or CMBs. Fatty liver was seen in 81.8% of classic (n = 9) vs 16.7% of variant AT (n = 3) and was significantly associated with the presence of CMBs (*p* < 0.01, OR 21.0, CI 1.856–250.8) but not WMHs (*p* = 0.358, OR 0.359, CI 0.056–2.189).

**Table:**
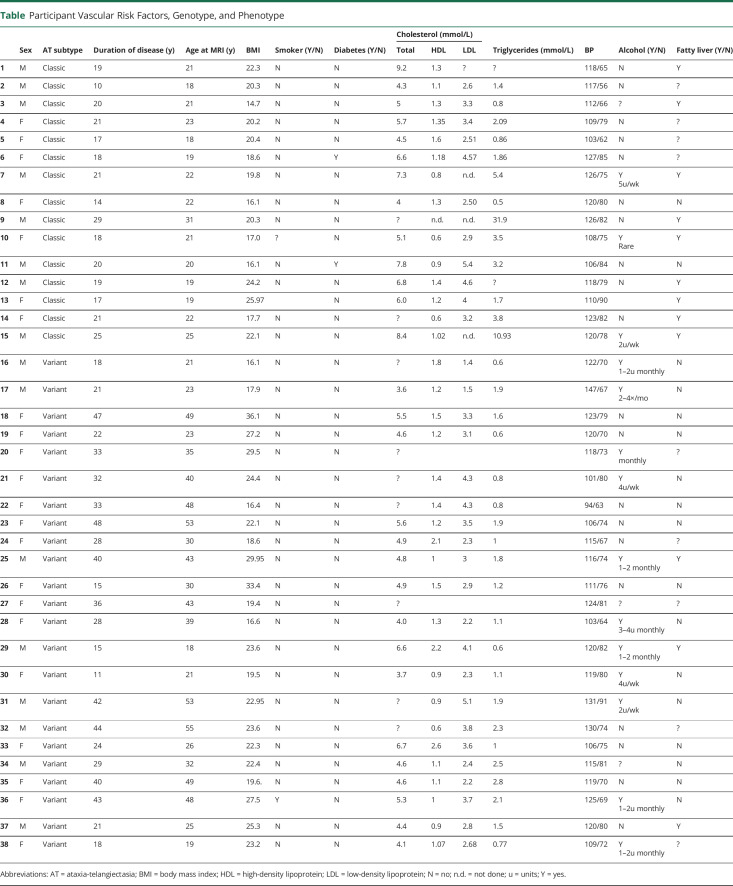
Participant Vascular Risk Factors, Genotype, and Phenotype

## Conclusions

We report that CMBs are common and sometimes extensive in classic AT but mainly extracerebellar. In contrast, individuals with milder variant AT had a higher proportion of WMHs with a lower incidence of CMBs. However, WMH burden was overall mild, and the mechanism is unknown.

It is possible that CMBs result from small vascular abnormalities, i.e., telangiectasia, such as those seen in the viscera. This is supported by the fact that ocular telangiectasia is usually present in classic AT, but not necessarily in variant patients. An alternative proposed mechanism of white matter edema in AT are transudates or exudates from leaky vessels.^5^ These leaky, more fragile vessels could predispose to hemorrhage. Previous autopsies found vascular abnormalities with spongy degeneration surrounding vessels in the cortex.^6^

WMHs and CMBs are markers of small vessel disease in other neurodegenerative disorders.^2^ As this is a retrospective analysis, we acknowledge the limitations of lack of control data. Further studies are required to understand the clinical implications and origins of CMBs. CMBs are a known risk factor for impaired cognitive function and increase in incidence with age.^2^ However, an incidence of 61% CMBs in young patients admitted with ICH has previously been reported.^7^

Clinicians need to be aware of the high proportion of CMBs in classic AT, which predisposes patients to ICH. Anticoagulation should ideally be avoided in these cases. Further studies will be required to evaluate the association between WMH, CMB, and AT neurodegeneration.
